# Prognostication in inflammatory bowel disease

**DOI:** 10.3389/fmed.2022.1025375

**Published:** 2022-10-06

**Authors:** Elizabeth A. Spencer, Manasi Agrawal, Tine Jess

**Affiliations:** ^1^Division of Pediatric Gastroenterology and Nutrition, Icahn School of Medicine, Mount Sinai Hospital, New York, NY, United States; ^2^Division of Gastroenterology, Icahn School of Medicine, Mount Sinai Hospital, New York, NY, United States; ^3^Center for Molecular Prediction of Inflammatory Bowel Disease, PREDICT, Aalborg University, Aalborg, Denmark; ^4^Department of Gastroenterology and Hepatology, Aalborg University Hospital, Aalborg, Denmark

**Keywords:** inflammatory bowel disease, Crohn's disease, ulcerative colitis, precision medicine, prognostication

## Abstract

Personalized care in inflammatory bowel diseases (IBD) hinges on parsing the heterogeneity of IBD patients through prognostication of their disease course and therapeutic response to allow for tailor-made treatment and monitoring strategies to optimize care. Herein we review the currently available predictors of outcomes in IBD and those on the both near and far horizons. We additionally discuss the importance of worldwide collaborative efforts and tools to support clinical use of these prognostication tools.

## Introduction

Inflammatory bowel disease (IBD) is a heterogeneous (1) and increasingly common (2, 3) disease. The past decade has brought hope in the form of a growing therapeutic armamentarium; however, even with this positive change, an imprecise, “one size fits all,” treatment algorithm is often applied to all patients leading to stagnation in gains in medication effectiveness and complication reduction. In this mini review, we strive to discuss the currently available and future methods to prognosticate in IBD; successful prognostication in IBD will encompass prediction of a patient's (1) disease course, (2) treatment response, and (3) risk of adverse effects or toxicities from therapy.

## Current predictors

**Figure 1:**
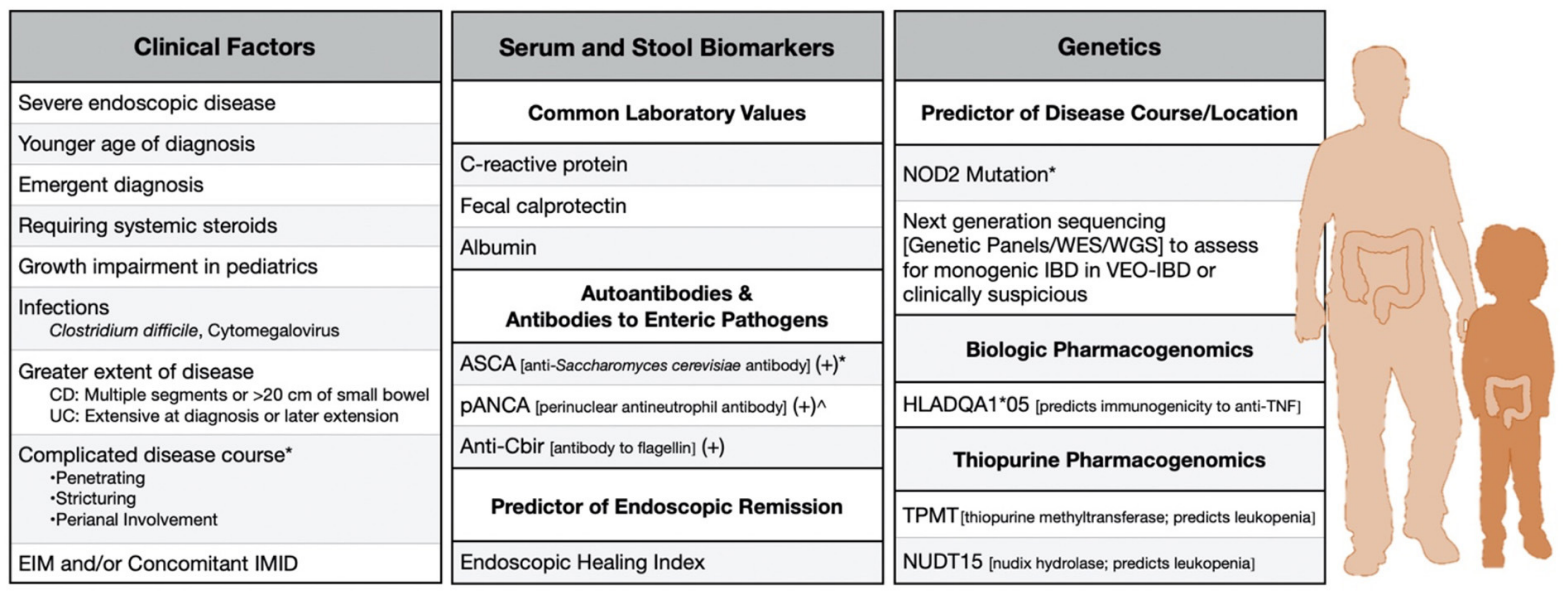
Clinically-available risk factors to predict severe disease in adult and pediatric IBD. *Factor in Crohn's Disease. ^∧^Factor in ulcerative colitis. CD, Crohn's disease; UC, ulcerative colitis; EIM, Extraintestinal manifestation; IMID, Immune-Mediated inflammatory disease.

In current clinical practice, most prognostication is rooted in clinical variables supplemented with traditional laboratory monitoring, like C-reactive protein (CRP) and fecal calprotectin (FC). Serologic antibody responses and pharmacogenomic tests to determine the safety of anti-TNF and thiopurine therapies can also be practically undertaken (Figure 1).

### Clinical features and disease course

Clinical factors have been identified in numerous retrospective analyses of both Crohn's disease (CD) and ulcerative colitis (UC) to predict disease course. These include age of onset for both diseases (4, 5), disease duration (6–8), disease extent and phenotype (9–11), extraintestinal manifestations (12), and concomitant immune mediated inflammatory disorders (13). Race, ethnicity, and sex have also been associated with differences in outcomes (14, 15), likely entangled with their complex intersectionality with social determinants of health (16). Additionally, cigarette smoking has long been known to be associated with complications and need for therapy escalation in CD (11). Unfortunately, the observational nature of the studies in which many of these predictors were identified limits their predictive performance and often precludes rigorous subgroup analyses (17).

### Clinically-available proteomics

C-reactive protein (CRP) and fecal calprotectin (FC) are commonly used markers to assess disease activity in IBD. An elevated CRP at diagnosis has been associated with a later need for surgery in both CD and UC (18). An elevated CRP can also portend an increased risk of hospitalization or resection in CD in the face of clinical remission (19). FC is more sensitive and specific for intestinal inflammation than CRP (20), and serial measurements have been shown to predict disease progression and/or relapse (21–23). A 13-protein panel, the endoscopic healing index (EHI), has been validated to predict endoscopic remission in CD and may be used in precision monitoring; this showcases the possible clinical utility of more comprehensive protein panels (24). However, CRP, FC, and EHI are all measures of active inflammation and are, thus, not truly predictive despite their obvious clinical usefulness.

Serologic markers that reflect immune responses against enteric pathogens and autoantigens, like perinuclear antineutrophil antibody (pANCA), anti-*Saccharomyces cerevisiae* antibody (ASCA), antibody to *Escherichia coli* outer-membrane porin C (OmpC), and antibody to flagellin (CBir1), have been tied to disease course. In a large prospective study of pediatric patients with CD, patients positive for two or more antimicrobial antigens progressed to complicated disease more rapidly than those with one or none (25). Beyond pANCA, granulocyte-macrophage colony-stimulating factor (GMCSF) is another autoantibody associated with disease course; high expression of GMCSF autoantibodies has been associated with stricturing and penetrating behavior in CD (26–28). The antimicrobial antibodies have been shown to rise prior to disease inception in PREDICTS (PRoteomic Evaluation and Discovery in an IBD Cohort of Tri-service Subjects), where pre-diagnosis serum from US army members was examined (29); since disease duration is closely tied with complication, it is unclear to what extent these antibodies serve as a proxy for disease duration and to what extent they give insight into the disease course.

### Genomics

Thiopurine methyltransferase (TPMT), which was identified in the 1980s as playing a critical role in thiopurine metabolism, has variants associated with decreased enzymatic activity that may result in severe leukopenia. It is currently recommended to check TPMT prior to thiopurine initiation in case a patient's genotype warrants lowering the dose or avoiding the medication altogether (30). Nudix hydrolase (NUDT15) mutations have similarly been implicated in leukopenia risk in European and Asian populations (31–34); a randomized trial (*n* = 118) demonstrated that adjusting dosing based on NUDT15 genotype significantly reduced leukopenia (35). In an illustration of the vital importance of pharmacogenomics in risk reduction, one study showed that the combination of TPMT and NUDT15 mutations accounted for ~50% of severe thiopurine-induced leukopenia (32). It is now recommended to check both prior to initiating thiopurine therapy. A polymorphism in the HLA class II region (rs2647087) is tied to pancreatitis in those on thiopurines, conferring a 17% pancreatitis risk in homozygotes, and it may warrant incorporation into a broader pharmacogenomic assessment (36).

There has been much work to examine if similar risk variants exist for biologic therapies. Most notably, the Personalizing Anti-TNF Therapy in CD (PANTS) consortium identified a variant, HLA-DQA1^*^05, associated with a significantly increased the risk of immunogenicity to anti-TNF (HR 1.90, 95% CI 1.60–2.25) in 1,240 biologic-naïve patients in the UK biobank (37). This has since been replicated in other cohorts, but the clinical action to take may be less straightforward as this risk variant did not confer risk for anti-drug antibody formation in two cohorts managed with proactively optimized infliximab monotherapy from induction (38, 39); this signals that those with this risk variant could likely equally be treated with combination therapy or early proactively optimized monotherapy. Further study and collaboration across diverse populations are needed to continue to study the role of pharmacogenomics in the many new therapies currently available for IBD.

Beyond pharmacogenomics, next generation sequencing, such as genetic panels or whole exome or genome sequencing, is available for situations where monogenic IBD may be suspected, such as in very early onset IBD (40). Beyond monogenic IBD, there have been numerous analyses leading to identification of a plethora of genetic variants that contribute to the development of IBD (41, 42). Studies naturally followed to examine if these risk variants were also tied to disease prognosis. A classic example of this are polymorphisms of nucleotide-binding oligomerization domain 2 (*NOD2*) that are involved in host-microbe immune responses; variants in NOD2 were the first-identified for CD and remain those that confer the greatest risk (43, 44). NOD2 mutations were initially noted to be associated with an increased risk of complicated CD and surgery (45). Yet, in a later large study, NOD2 was noted to be strongly associated with ileal disease location, and, when accounting for this confounding by phenotype, there no longer remained an association with disease course (46).

## Future predictors

There is much on the horizon with significant research, increasingly with an eye toward global, collaborative efforts, being performed in the space of prognostication (Box 1). Predictors either incorporate already existing factors, like those discussed above, with novel predictors to form clinical decision support tools or can be completely novel, like those in the glycomics, more expansive genomics (e.g., polygenic risk scores), epigenetics, gene expression, and microbiomics.

Box 1 **Large predictive biomarker studies in IBD.**

**Multiomic projects**

- IBD Multiomics database: Multiomic profiling project of 90 participants over the course of 1 year
- IBD Plexus: Interconnected exchange platform of Crohn's and Colitis Foundation with various purposes, including biomarker identification and hypothesis validation
- IMI (Innovative medicines initiative): Identification of the molecular mechanisms and tissue signatures of non-response to treatments, relapses and remission in autoimmune, inflammatory and allergic conditions
- PMI (Precision Medicine in Chronic Inflammation): Working to develop molecular tools for treatment of chronic inflammatory disease
- PREDICT (DK): Cohort of 10,000 IBD and 10,000 healthy individuals with creation of a data lake incorporating a plethora of multi'omic data with a wealth of clinical data in the setting of a longitudinal nationwide register data aimed at addressing biological mechanisms and heterogenous course of IBD with an eye at precision, encompassing prevention and prognostication
- PROTECT (Predicting Response to Standardized Pediatric Colitis Therapy) cohort: Prospective study of treatment-nanewly diagnosed pediatric patients with UC
- RISK (Risk Stratification of Rapid Disease Progression in Children with Crohn's Disease) cohort: Prospective study of treatment-nanewly diagnosed pediatric patients with CD
- SYSCID (Systems medicine approach to chronic inflammatory diseases) Consortium: Aim to identify core disease signatures, shared and/or unique, of chronic inflammatory diseases using systems-level, and multi'omic techniques

**Proteomic projects**

- Collaborative IBD Biomarker Research Initiative (COLLIBRI): Aim to identify novel insights and biomarker signatures of IBD, currently with a focus on proteomics
- IBD-CHARACTER: A proteomic biomarker discovery study of 400 patients with newly diagnosed, treatment-naive IBD, 200 symptomatic patients without evidence of IBD, and 200 healthy age-matched controls; seeking to identify proteomic markers associated with clinical outcomes
- Nurses' Health Study: Biorepository study (United States nurses, enrollment of women) aiming to identify novel serum biomarkers particularly before development of IBD
- PREDICTS (PRoteomic Evaluation and Discovery in an IBD Cohort of Tri-service Subjects): Biorepository study (United States military personnel, primarily men) aiming to identify novel serum biomarkers particularly before development of IBD

**Transcriptomic projects**

- IBD Transcriptome and Metatranscriptome Meta-Analysis (TaMMA) platform: comprehensive survey of publicly available RNA-seq datasets from IBD-derived and control samples across different tissues
- PROFILE (PRedicting Outcomes For Crohn's dIsease using a moLecular biomarker) trial: Biomarker-stratified trial in patients with newly diagnosed Crohn's disease using the PredictSURE IBD

**Metabolomic and microbiomic projects**

- IBD-RESPONSE: Prospective study of genetic and metagenomic markers of response to biological and Janus kinase inhibitor therapy in IBD
- PREdiCCt (The PRognostic effect of Environmental factors in Crohn's and Colitis): An observational study aiming to recruit 3,100 patients with IBD in remission, seeking to determine environmental factors—including contributions from dietary intake and the gut microbiome—to both remission and relapse of inflammation

**Genetic projects**

- IBD Bioresource: Observational United Kingdom study aiming to further understand the functional effect of IBD-associated gene variants
- PANTS (The Personalized ANti-TNF therapy in Crohn's disease Study): Observational study aiming to provide novel insights into anti-TNF response and non-response

### Proteomics

Numerous studies are currently on-going examining the use of broader panels of proteins, which may allow for improved precision. IBD Character, which is a prospective case-control study assessing the utility of proteomics in prognostication, presented the results from an inception cohort of 328 patients with IBD that identified 5 proteins (ITGAV, EpCAM, IL18, SLAMF7, and IL8) that could distinguish a high-risk group, defined as those who needed biologic agents or surgery after a period of remission, but this panel still needs prospective validation (47).

### Glycomics

It is becoming increasingly clear that glycosylation plays a role in IBD from disease inception to progression. Generally, abnormal glycosylation has been linked to aberrations in homeostasis that can lead to inflammation (48). Glycomic markers are currently being studied for their potential to be used in assessing prognosis and treatment response, and, in fact, decreased galactosylation has been correlated with more severe CD and UC (49). They may also facilitate our assessment of treatment response, glycoprotein acetylation (GlycA) has also been associated with achieving mucosal healing in patients with CD and UC (50, 51).

### Genetics

Despite the current, pharmacogenomic-focused clinical use of genetics described above, familial studies, where affected relatives often share similar courses of disease, still implicate a genetic contribution to prognosis (52). This provoked the question of possible contribution of a separate set of alleles that modify disease course. This was explored in a large (>2,700) genome-wide association study (GWAS). First, they created a score from a traditional set of 170 CD susceptibility loci, and this was intriguingly not associated with prognosis. They then went on to identify four unique loci, not linked to susceptibility to IBD, that were associated with poor prognosis (*FOXO3, XACT*, a region upstream of *IGFBP1*, and a large swath of the MHC); this suggests that unique scores may need to be created for prediction of disease and prediction of disease course (53). While a similar study has yet to be performed in UC, the HLA type DRB1^*^0103, found also in the MHC region, has been associated with extensive disease and colectomy risk, speaking to a potential sharing of variants associated with prognosis between CD and UC (54).

Polygenic risk scores (PRS), which sum the risk of all alleles weighted by effect size and include those that would fall below the normal standards for GWAS significance, are gaining interest. They have been found to provide improved risk assessments in other fields, like oncology and cardiology (55). Within the RISK (Risk Stratification of Rapid Disease Progression in Children with CD) cohort, an inception cohort of 913 pediatric patients with CD, neither a PRS nor NOD2 was associated with complicated behavior (25). Yet, methods used to create these PRS vary widely, which may have impacted these findings. Research on the use of PRS may be enhanced both by rapidly improving techniques as well as by a focus on pharmacogenomics and personalized care in other fields which will improve access to large, diverse genetic datasets (55). Gettler et al. has shown that genetic association data from diverse populations improves IBD prediction across all populations, showcasing the importance of including varied populations (56).

### Transcriptomics

There have been numerous important studies over the last 5 years which have identified transcriptional signatures tied to prognosis (25, 57–60). In a 2017 study of patients with IBD, an oncostatin M (OSM)-associated inflammatory transcriptomic module was noted to be associated with response to anti-TNF therapy (58). In that same year, RISK identified an extracellular matrix tissue transcriptomic signature at diagnosis that could predict stricturing in 3 years of follow-up; they then combined this with other traditional clinical and serologic markers to make a prognostic risk model that still requires validation (25). These findings have inspired further interest in supporting large prospective cohorts and working collaboratively to identify and then validate these transcriptional signatures.

Marked advances over the last 5 years of single-cell sequencing technologies have allowed for high resolution mapping of the intestinal cellular landscape. This type of single-cell analysis was recently performed in a cohort (*n* = 22) of adult patients with CD and identified a transcriptomic module from cells derived from the lamina propria; this module was found to be associated with failure to achieve durable corticosteroid-free remission in the RISK cohort (59), speaking to the potential for generalizable, reproducible data from single-cell sequencing.

PredictSURE IBD (PredictIMMUNE, Cambridge, UK) is a CD8+ T cell gene expression profiling panel, validated to prognosticate IBD patients into low- and high-risk (60). The prognostic value of the test was also noted to be affected by steroid use, which may dictate the timing of the test, limiting its clinical use (61). Due to methodologic issues, this signature could initially not be found in validation cohorts of pediatric and adult patients (62); upon follow-up, methodologic review, the signature could, in fact, be identified in both cohorts (63). It represents an intriguing clinical application of transcriptomics and presents an instructive tale of the complexity of validating an advanced ‘omic test for clinical use.

### Microbiomics

The microbiome plays a critical role in the pathogenesis of IBD, and it follows that studies would investigate how the microbiome may affect disease course. This is further supported by the studies showing that complicated disease is associated with increased serologic responses to enteric pathogens (29), with the earlier noted caveat that these responses rise with increasing disease duration. Mouse models are also instructive, showing that stool from patients with colitis can exacerbate mouse models of colitis (64). However, parsing the changes in microbiome from myriad changes in the environment (e.g., diet and smoking status) makes translation of the microbiome changes into an actionable prognostic factor complicated. There is work being performed in this area to unravel the contributions of the microbiome (Box 1).

### Metabolomics

Numerous metabolomic profiles have been associated with prognosis and treatment response in IBD (65). However, as with the other mentioned biomarkers, prospective studies are scarce. In a small (*n* = 20), prospective pilot study of patients with UC, Keshteli et al. described urinary and serum metabolites in a 12-month period. When compared with patients still in remission, patients that relapsed had significantly higher levels of *trans*-aconitate in urine and 3-hydroxybutyrate, acetoacetate, and acetone in serum as well as lower levels of acetamide and cystine in urine (66). A further prospective study, also in UC, showed that histidine levels were predictive of relapse within 1 year (67). Further study on these profiles needs to be performed before they can be incorporated into clinical practice.

### Integrating multi'omic data

There are a number of cohorts (Box 1) aimed at combining multi'omic data to better characterize such a heterogenous disease. PREDICT (DK) is a leading example of this; it pairs superb nationwide register data that allows for detailed, longitudinal clinical information with a wealth of data including genetics, epigenetics, antibodies, inflammatory markers, metabolomics and microbiomes on thousands of patients. This data lake of clinical information and multi'omic information is expected to yield new insights into precision (68). Deep, longitudinal multi'omic profiling has been performed in other chronic diseases, such as diabetes mellitus, with this profiling allowing for the prediction of disease course and complications, such as insulin resistance (69), but much of this rich, long-term profiling has been done in pre-disease-inception, high-risk cohorts in IBD (70, 71) and other IMIDs (72). Generally, network-based methods will be critical in integrating multi'omic data and interrogating the IBD “interactome” (73, 74); these network analyses lead to insight into gene regulatory networks, protein-protein interactions, and microbiome-metabolomic networks (75–77), and they could potentially be utilized to identify a patient's disease subtype and optimal therapeutic target(s).

## Incorporation in the clinic

An issue that plagues these predictors is the difficulty in implementation in the clinic. Some of this lies in the need to educate providers on understanding the limitations of the predictors. False positives and negatives are common with any such predictive test, and this possibility needs to be understood by providers and well-explained to patients. However, this concept can be difficult to communicate well, particularly in short clinical visits. Provider education must seamlessly accompany these clinical tests, and leveraging electronic medical records to prompt providers to utilize these prognostic tests at diagnosis may be helpful (78). Clinical decision support tools (CDSTs) can also aid clinicians in incorporating predictors. A small number of CDSTs have been created, typically incorporating data on medication clearance, patient-reported outcomes, and lab values to predict remission or response; one such CDST for IFX achieved an accuracy of 80% in predicting endoscopic healing (79–81). As with all of the above tests, prospective validation of CDSTs must be performed before incorporation into routine clinical care.

Another barrier to clinical implementation is the complexity of reimbursement; precision testing often does not align with traditional payment schemes as precision care by its very nature requires more upfront costs for a theoretical, long-term pay off. It has been postulated that payment systems and data collection would need to be overhauled to allow for precision integration in clinics across the world (82).

## Incorporation in clinical trials

A critical use of precision medicine is within clinical trials to help in patient selection. This has been extensively used in oncologic therapies (83), but it is also now entering the IBD space. The EXPLORER trial (NCT02764762), an open-label Phase 4 trial using triple combination therapy with vedolizumab, adalimumab, and methotrexate, used one of two prognostic tools [1. CD-PATH: includes clinical characteristics, genetics (NOD2), and serologic markers (84) or 2. AGA Clinical Care Pathway: includes clinical characteristics (85)] to identify biologic-naive patients with CD at moderate-to-high risk of complication to include in the study (86). The PROFILE (Predicting Outcomes for Crohn's Disease using a Molecular Biomarker) Trial (ISRCTN11808228) is randomizing patients to variably aggressive therapeutic strategies with patients defined a priori into low- and high-risk subgroups based on the PredictSURE IBD test (87). These two trials are trailblazers, illustrating how innovation in trial design is a cornerstone of precision care and can address the issue of patient heterogeneity in IBD; future trials should learn and build from their protocols.

## Collaboration

Shared definitions and clear methodologies are critical to allow for collaborative and reproducible research; the Scientific Workshop Steering Committee for the European Crohn's and Colitis Organization (ECCO) published an excellent statement on Precision Medicine in IBD with proposed universal outcomes for prognostic biomarkers to bridge research between siloed academic centers across the world (88). As an example, the IBD Transcriptome and Metatranscriptome Meta-Analysis (TaMMA) framework pulled together all the publicly available IBD RNA-sequencing datasets (89); such aggregate data would be more easily feasible if metadata and outcomes were standardized and methodologies were clearly presented and consistent within the IBD space. Recognition of a need for data harmonization across academic centers given an inherent alignment of goals to best serve a growing and heterogeneous patient population is just the first step in achieving practice-changing collaborations to support precision medicine; emphasis needs to continue to shift from research of individuals to research from the IBD community at large.

## Conclusion

While there are currently feasible tools available to prognosticate in IBD, it remains a space ripe for collaborative research efforts to deliver personalized care using rich multi-omic data to improve outcomes in patients with IBD.

## Author contributions

ES: concept, literature search, drafting of manuscript, and critical revision of the manuscript for important intellectual content. MA and TJ: concept and critical revision of the manuscript for important intellectual content. All authors contributed to the article and approved the submitted version.

## Funding

ES and MA are supported by grants from the NIH (ES: K23DK125760-01 and MA: K23DK129762-02). TJ was supported by a National Center of Excellence grant from the Danish National Research Foundation (DNRF148).

## Conflict of interest

Author ES has served as an advisory board member for Prometheus Laboratories. The remaining authors declare that the research was conducted in the absence of any commercial or financial relationships that could be construed as a potential conflict of interest.

## Publisher's note

All claims expressed in this article are solely those of the authors and do not necessarily represent those of their affiliated organizations, or those of the publisher, the editors and the reviewers. Any product that may be evaluated in this article, or claim that may be made by its manufacturer, is not guaranteed or endorsed by the publisher.
